# Anti-inflammatory effects of ursodeoxycholic acid by lipopolysaccharide-stimulated inflammatory responses in RAW 264.7 macrophages

**DOI:** 10.1371/journal.pone.0180673

**Published:** 2017-06-30

**Authors:** Wan-Kyu Ko, Soo-Hong Lee, Sung Jun Kim, Min-Jae Jo, Hemant Kumar, In-Bo Han, Seil Sohn

**Affiliations:** 1 Department of Neurosurgery, CHA University, CHA Bundang Medical Center, Seongnam-si, Gyeonggi-do, Republic of Korea; 2 Department of Biomedical Science, CHA University, Seongnam-si, Gyeonggi-do, Republic of Korea; National Institutes of Health, UNITED STATES

## Abstract

**Purpose:**

The aim of this study was to investigate the anti-inflammatory effects of Ursodeoxycholic acid (UDCA) in lipopolysaccharide (LPS)-stimulated RAW 264.7 macrophages.

**Methods:**

We induced an inflammatory process in RAW 264.7 macrophages using LPS. The anti-inflammatory effects of UDCA on LPS-stimulated RAW 264.7 macrophages were analyzed using nitric oxide (NO). Pro-inflammatory and anti-inflammatory cytokines were analyzed by quantitative real time polymerase chain reaction (qRT-PCR) and enzyme-linked immunosorbent assay (ELISA). The phosphorylations of extracellular signal-regulated kinase (ERK), c-Jun N-terminal kinase (JNK), and p38 in mitogen-activated protein kinase (MAPK) signaling pathways and nuclear factor kappa-light polypeptide gene enhancer in B-cells inhibitor, alpha (IκBα) signaling pathways were evaluated by western blot assays.

**Results:**

UDCA decreased the LPS-stimulated release of the inflammatory mediator NO. UDCA also decreased the pro-inflammatory cytokines tumor necrosis factor-α (TNF-α), interleukin 1-α (IL-1α), interleukin 1-β (IL-1β), and interleukin 6 (IL-6) in mRNA and protein levels. In addition, UDCA increased an anti-inflammatory cytokine interleukin 10 (IL-10) in the LPS-stimulated RAW 264.7 macrophages. UDCA inhibited the expression of inflammatory transcription factor nuclear factor kappa B (NF-κB) in LPS-stimulated RAW 264.7 macrophages. Furthermore, UDCA suppressed the phosphorylation of ERK, JNK, and p38 signals related to inflammatory pathways. In addition, the phosphorylation of IκBα, the inhibitor of NF-κB, also inhibited by UDCA.

**Conclusion:**

UDCA inhibits the pro-inflammatory responses by LPS in RAW 264.7 macrophages. UDCA also suppresses the phosphorylation by LPS on ERK, JNK, and p38 in MAPKs and NF-κB pathway. These results suggest that UDCA can serve as a useful anti-inflammatory drug.

## Introduction

Inflammatory response is a physiological process against detrimental stimuli such as pathogens in our bodies [1] and macrophages play a critical role in inflammatory responses through the production of various cytokines [2]. Activated macrophages respond to pathogen invasion by releasing various pro-inflammatory cytokines including tumor necrosis factor-α (TNF-α), interleukin 1-α (IL-1α), interleukin 1-β (IL-1β), interleukin 6 (IL-6) [3, 4], and inflammatory mediators such as nitric oxide (NO) [5, 6]. However, excessive inflammatory response can lead to many diseases such as atherosclerosis [7], rheumatoid arthritis [8], and asthma [9]. Thus, various studies associated with the inhibition of excessive inflammatory responses have been reported over the past several decades [10–12].

Ursodeoxycholic acid (UDCA), used as a Chinese medicine for more than 3000 years [13], has been studied for its alleviating effects on atherosclerosis, diabetes, and renal disease [14]. UDCA was originally approved by the US Food and Drug Administration (FDA) for the treatment of several cholestatic liver disorders based on its choleretic effects to protect hepatocytes from hydrophobic bile acids [15–17]. However, to the best of our knowledge, only one study conducted an evaluation of UDCA as an anti-inflammatory drug in a lipopolysaccharide (LPS)-stimulated inflammatory process model [18]. LPS is the major component of the outer membrane of gram-negative bacteria, a type of bacteria which is frequently utilized in *in vitro* studies to induce an inflammatory response [19]. Joo et al. investigated the anti-inflammatory effects of UDCA through the NO test and with IL-1β. Based on their findings, they suggested that UDCA could inhibit the inflammatory process of microglial cells [18]. However, studies with only NO and IL-1β expression levels may not sufficiently support the authors’ claims.

In this study, we aim to evaluate the anti-inflammatory effects of UDCA further through an analysis of certain inflammatory and anti-inflammatory pathways.

## Materials and methods

### Preparation of UDCA and LPS

The UDCA was obtained from TCI (Tokyo Chemical Industry Co., LTD, Japan) and solubilized in Dulbecco’s modified eagle medium (DMEM, GIBCO, Grand Island, NY) containing 10% fetal bovine serum (FBS, GIBCO) and 1% penicillin-streptomycin (PS, GIBCO) for each experimental concentration. LPS was purchased from Sigma Aldrich (Sigma, St. Louis, MO) and melted with distilled water (100 μg/ml), after which it was diluted with DMEM (1 μg/ml) to induce an inflammatory response in RAW 264.7 macrophage cells [20].

### Experimental group

We compared the differences among 4 groups; There are a UDCA treated group, a LPS only treated group (LPS group), a LPS containing UDCA treated group (UDCA group), and a no treatment group in RAW 264.7 macrophages (control group).

### Cell culture

The murine macrophage cell line, RAW 264.7, was obtained from the Korean Cell Lines Bank and cultured in DMEM containing 10% FBS and 1% PS in a humidified 5% of CO_2_ atmosphere. The cells were incubated on the 100-mm culture plate (Falcon Becton Dickinson, Lincoln Park, NJ) and sub cultured when were fluent with 90%. The cells were washed with fresh media and changed with other fresh media daily.

### Cell viability assay

The cell viability in various density of UDCA (0, 0.5, 1, 2, 5 mM) was evaluated using a cell counting kit (CCK-8, Dojindo Molecular Technologies Inc., Japan) and a live/dead staining kit (Invitrogen Life Sciences, Carlsbad, CA, USA). After seeding the cells in 48-well culture plate (Falcon, 2 × 10^4^ cells/well, n = 3 per group), the adhering and proliferating cells were measured. At 24 h, cells were washed with Dulbecco's phosphate-buffered saline (DPBS, Invitrogen). After the addition of fresh media containing CCK-8 (500 μL of 0.1 mL/ml), the cells were incubated for 2 h.

After incubation, the intensity was measured by a microplate reader (Bio-Rad, Hercules, CA, USA) at a wavelength of 450 nm. The absorbance of the control group was fixed at 100% and the absorbance levels of other groups were calculated relative to that level. Under the same conditions, cells were stained with calcein-AM/ethidium homodimer-1 (EthD-1) from live/dead staining kit. After reacting for 15 min, the cells in all groups were observed at 40 x magnification using an inverted fluorescence microscope (Olympus IX71, Japan).

### NO assay

The cells in the UDCA with or without LPS group (1 × 10^5^ cells/well, n = 3 per group) in a 96-well plate (Falcon) were pretreated with various concentrations of UDCA (0, 0.5, 1, or 2 mM) for 1 h and UDCA containing LPS groups stimulated with LPS (1 μg/mL) containing equal concentrations of UDCA for an additional 1, 6, or 24 h. The accumulated NO in the culture supernatant was detected using a Griess Reagent System (Promega, Madison, WI). Briefly, an equal volume of supernatant and sulfanilamide in a solution was mixed and incubated for 10 min at room temperature, after which a solution of naphthylethylenediamine dihydrochloride was added. The mixture was incubated for an additional 5 min, and its absorbance was measured at 548 nm using a microplate reader (Bio-Rad). The NO concentration in the supernatants was determined from a standard curve generated with sodium nitrite.

### RNA extraction and quantitative real-time polymerase chain reaction (qRT-PCR)

RAW 264.7 macrophages (2 × 10^5^ cells/plate) were seeded on a 35-mm culture plate (Falcon) and proliferated with UDCA, LPS or LPS containing UDCA. The cells in the UDCA with or without LPS group were pretreated with 1 mM of UDCA for 1 h before the zero time point. At the predetermined time points, seeded cells of total RNA were extracted using Trizol reagent (Invitrogen) according to the manufacturer’s instructions. Complementary DNA (cDNA) was synthesized from 1 μg of total RNA using a cDNA using synthesis kit (TAKARA, Shiga, Japan). The qRT-PCR was performed using an ABI Step One Real-time PCR System (Applied Biosystems, Warrington, UK) and a reaction mixture that consisted of SYBR Green 2 × PCR Master Mix, a cDNA template, and forward and reverse primers. The PCR protocol consisted of 40 cycles of denaturation at 95°C for 15 sec, followed by 60°C for 30 s to allow for extension and amplification of the target sequence. The relative expression levels of TNF-α, IL-1α, IL-1β, IL-6, and IL-10 were normalized to that of glyceraldehyde 3-phosphate dehydrogenase (GAPDH) using the 2-ΔΔCT method. The primers were obtained from Bioneer (Daejeon, Korea). The primer sequences used in this study are shown in Table 1.

**Table 1:** Nucleotide sequences of primers used in real-time qRT-PCR.

Gene	Forward (5’–3’)	Reverse (5’–3’)
TNF-α	AGCAAACCACCAAGTGGAGGA	GCTGGCACCACTAGTTGGTTGT
IL-1α	TTGGTTAAATGACCTGCAACA	GAGCGCTCACGAACAGTTG
IL-1β	AGTTGACGGACCCCAAAAG	AGCTGGATGCTCTCATCAGG
IL-6	GCTACCAAA CTGGATATAATCAGGA	CCAGGTAGCTATGGTACTCCAGAA
IL-10	CAGAGCCACATGCTCCTAGA	TGTCCAGCTGGTCCTTTGTT
GAPDH	AGGTCATCCCAGAGCTGAACG	CACCCTGTTGCTGTAGCCGTAT

### Enzyme-linked immunosorbent assay (ELISA)

The cells in the UDCA with or without LPS group (1 × 10^5^ cells/well, n = 3 per group) in a 96-well plate (Falcon) were pretreated with 1 mM UDCA for 1 h. LPS containing UDCA group stimulated with LPS (1 μg/mL) containing 1 mM UDCA for an additional 1, 6, or 24 h. TNF-α, IL-1α, IL-1β, IL-6 and IL-10 were detected under the most optimistic conditions. In brief, at the predetermined time points, accumulated proteins in supernatant fluid was detected in the 96-well plate of ELISA kits (Koma Biotech, Seoul, South Korea) and each absorbance was measured at 548 nm using a microplate reader (Bio-Rad).

### Western blotting

RAW 264.7 macrophages (2 × 10^6^ cells/plate) were seeded onto a 100-mm culture plate (Falcon) and proliferated with UDCA, LPS or LPS containing UDCA. The cells in the UDCA with or without LPS groups were pretreated with 1 mM of UDCA for 1 h before the zero time point. At 24 h, the cells were gently scraped with a cell scraper (SPL, Seoul, Korea) and collected in e-tubes. The collected cells were lysed by the addition of cold RIPA lysis buffer containing 2.5% deoxycholic acid, 0.5 M Tris-HCl, at pH 7.4, and with 1.5 M NaCl, 10% NP-40, and 10 mM EDTA (Sigma) with protease (Roche Applied Science, Indianapolis, IN) and phosphatase inhibitor cocktails (Sigma) in an ice box for 30 min, after which they were centrifuged at 13,000 rpm for 10 min. The concentration of protein was measured using a micro-plate spectrophotometer (Bio-Rad) at 595 nm. In this case, 40 μg of resolved equal amounts of proteins were congregated to 10% of sodium dodecyl sulfate polyacrylamide gel electrophoresis and transferred to nitrocellulose transfer membranes (Protran, Whatman, Germany). The membranes were incubated with 5% of skim milk for 1 h to block the nonspecific binding and then probed with the primary antibodies of phosphorylated forms of extracellular signal-regulated kinase (p-ERK, 1:1000), c-Jun N-terminal kinase (p-JNK, 1:1000), p38 (p-p38, 1:1000) and nuclear factor kappa-light polypeptide gene enhancer in B-cells inhibitor, alpha (p-IκBα, 1:1000). Subsequently, equal membranes were stripped and reprobed with the total forms of ERK (t-ERK, 1:1000), JNK (t-JNK, 1:1000), p38 (t-p38, 1:1000), and IκBα (t-IκBα, 1:1000). All the primary antibodies were purchased from Cell Signaling Technology (Danvers, MA, USA) except β-actin (1:2000, Abcam, Cambridge, UK). As an internal control, β-actin also probed in the membranes. All primary markers were followed by incubation with secondary antibodies (1:5000, Santa Cruz Biotechnology, Dallas, TX). The visualized signal bands were detected using a horseradish peroxidase procedure with a ChemiDoc XRS System (Bio-Rad). The phosphorylated form/total form (p/t form) volumes per the predetermined time points were calculated and quantified using the volume measurement program in ChemiDoc (Bio-Rad). The control group (0 h) of the p/t form volume was set at 1-fold and the ratio of the normalized fold change was also relatively calculated and quantified for 24 h.

### Statistical analyses

All values were presented as the mean ± standard deviation (SD). A one-way analysis of variance (ANOVA) followed by post-hoc tests were used to verify statistically differences among the groups. The differences with p-values for which *p < 0.05 and **p < 0.01 were considered statistically significant.

## Results

### Cytotoxicity of UDCA in RAW 264.7 macrophages

**Fig 1:**
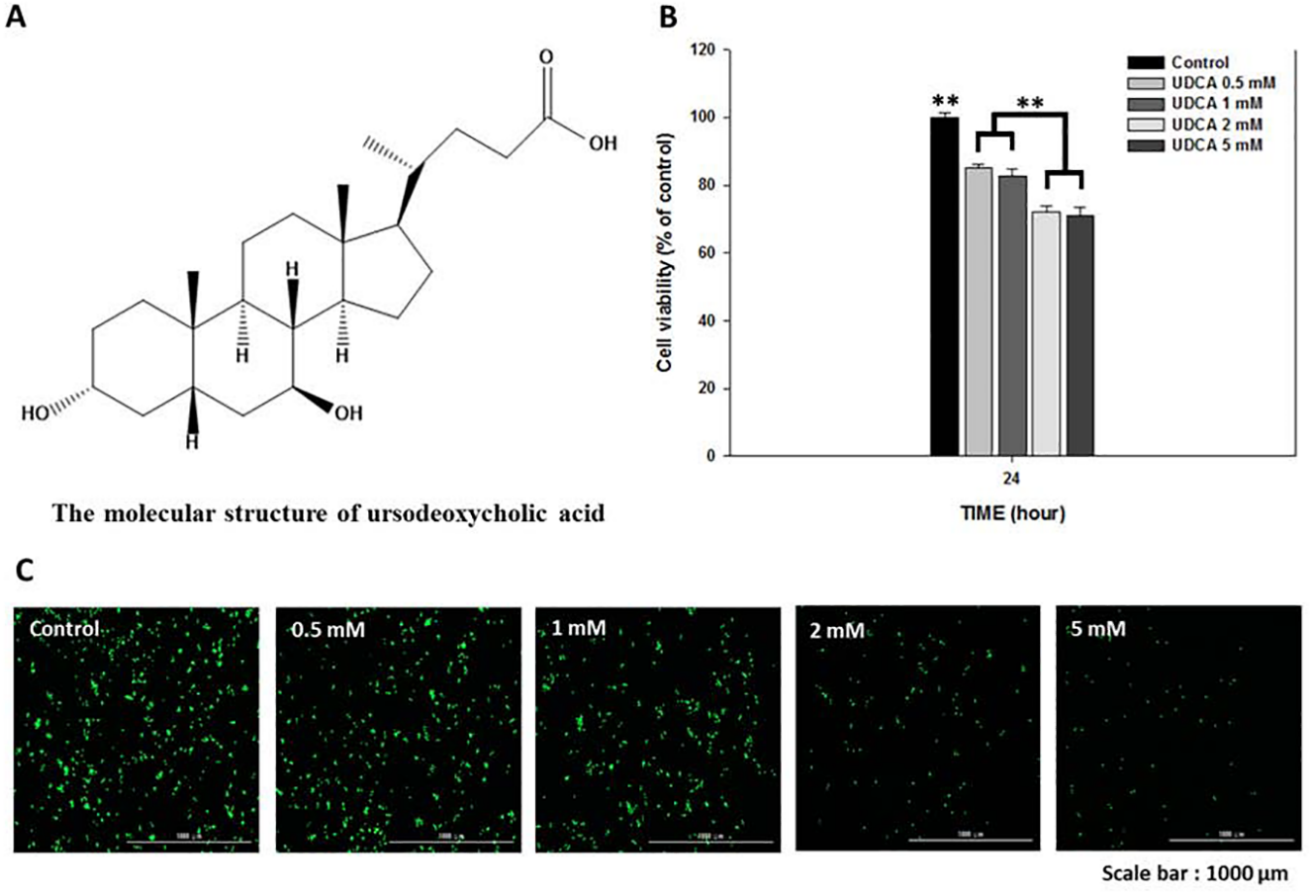
The molecular structure of ursodeoxycholic acid (UDCA) and the effect on the cell viability of RAW 264.7 macrophages. (A) The molecular structure of UDCA. The macrophages were treated with various concentrations of UDCA (0, 0.5, 1, 2, or 5 mM) for 24 h. (B) Cell viability was measured using a CCK-8 assay and (C) a live/dead staining kit. Results are mean ± SD of triplicate experiments: **p < 0.01, significant difference as compared to the control and to each other.

Fig 1A depicts the molecular structure of the UDCA [21]. Cell viabilities above 80% were noted in the 0.5 and 1 mM UDCA groups for 24 h in RAW 264.7 macrophages (Fig 1B, 85.27% ± 0.98 and 82.78% ± 2.08, respectively). However, cell viability levels in the 2 mM and 5mM UDCA groups were under 80% (72.15% ± 1.71 and 71.18 ± 2.25, respectively). In addition, the live/dead staining assay for 24 h in the Raw 264.7 macrophage cells (Fig 1C) showed a cell viability trend similar to that of the results shown in Fig 1B.

### Effect of UDCA on LPS-stimulated NO secretion

**Fig 2:**
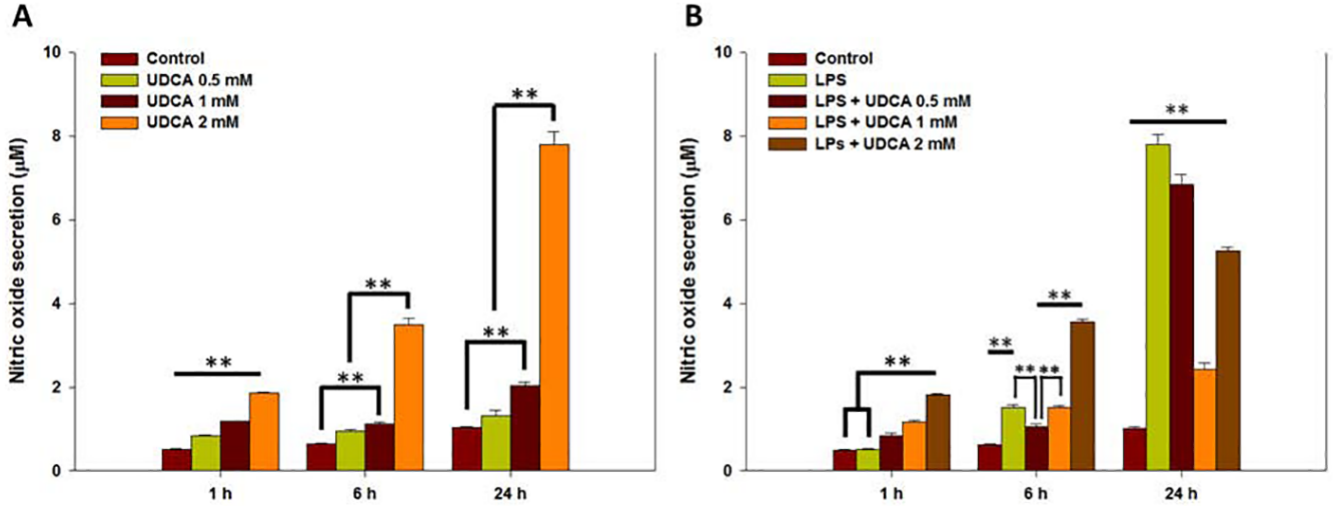
Nitric oxide (NO) secretion in RAW 264.7 macrophages treated with various concentrations of UDCA, lipopolysaccharide (LPS) or LPS containing various concentrations of UDCA. (A) The macrophages were treated with 0.5, 1, or 2 mM UDCA for 1, 6, or 24 h. (B) The macrophages of LPS alone group were treated with 1 μg/mL LPS for 1, 6, or 24 h. The macrophages of the LPS containing UDCA groups were pre-treated with 0.5, 1, or 2 mM UDCA for 1 h. Then treated with LPS (1 μg/mL) containing equal concentration of UDCA for an additional 1, 6, or 24 h. The collected supernatants were reacted with Griess reagent, and the absorbance level was measured at 548 nm. Results are mean ± SD of triplicate experiments: *p < 0.05 and **p < 0.01, significant difference as compared to the control and to each other at the time point of 1, 6, or 24 h.

NO of 0.5 and 1 mM UDCA groups increased slightly for 24 h. However, NO of the 2 mM UDCA group markedly increased at 24 h (7.79 ± 0.30, Fig 2A). NO of the LPS group increased steadily for 24 h, with a maximum index at 24 h (7.81 ± 0.24, Fig 2B). However, the NO of various concentrations of UDCA containing LPS groups decreased significantly compared to that of the LPS alone group in the order of 0.5 (6.85 ± 0.24), 2 (5.26 ± 0.08), and 1 mM (2.42 ± 0.15) at 24 h (Fig 2B). In other words, 1 mM UDCA showed the lowest NO level at 24 h.

### Effect of UDCA on inflammatory and anti-inflammatory cytokines in mRNA levels in LPS-stimulated RAW 264.7 macrophages

**Fig 3:**
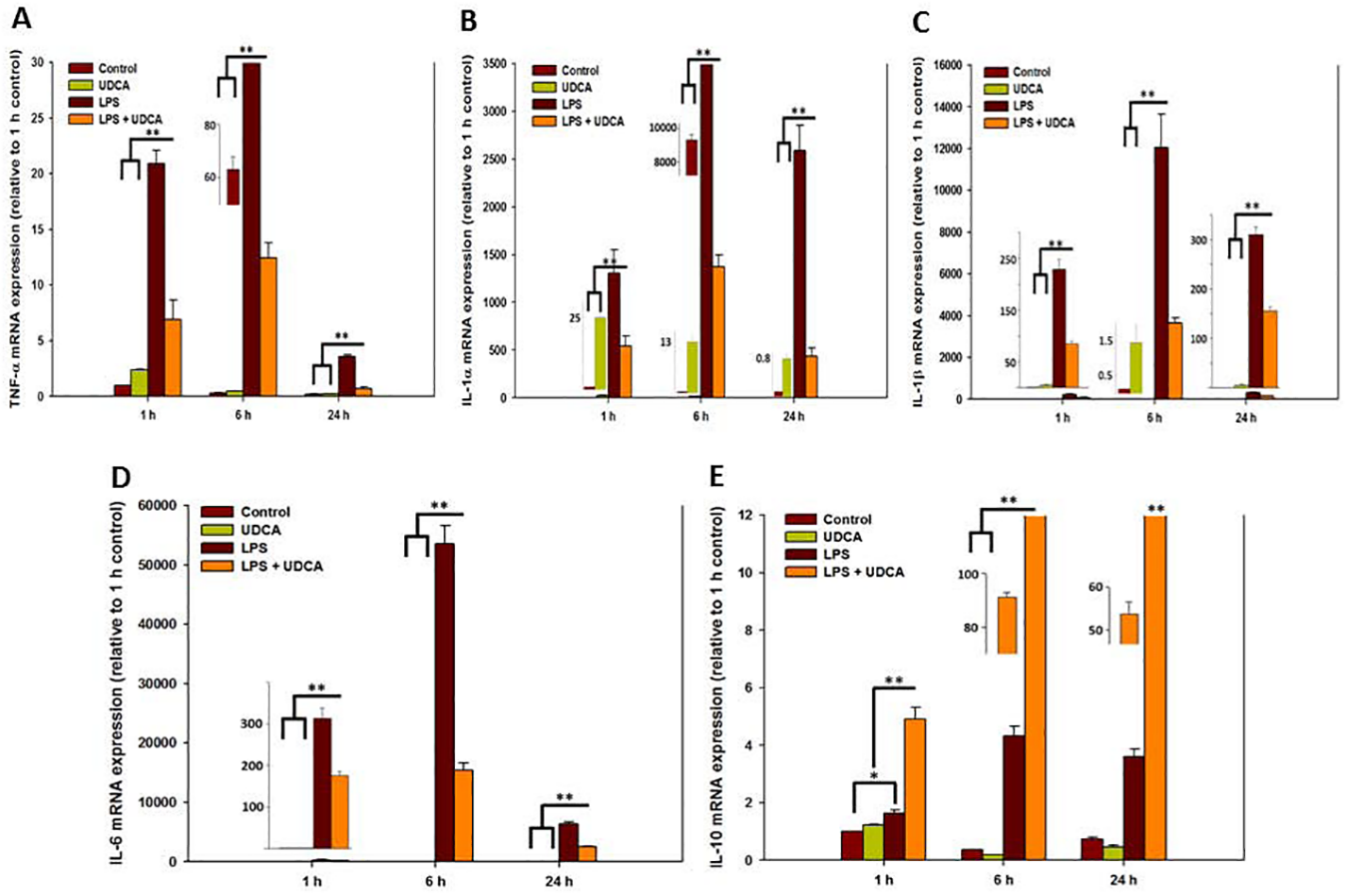
The mRNA expression in RAW 264.7 macrophages treated with UDCA (1 mM), LPS (1 μg/mL), or LPS (1 μg/mL) containing 1 mM UDCA for 1, 6, or 24 h. The macrophages in the UDCA with or without LPS group were pretreated with 1 mM UDCA for 1 h before the zero time point. Then, the UDCA alone group treated with 1 mM UDCA for an additional 1, 6, or 24 h and UDCA containing LPS group treated with LPS (1 μg/mL) containing 1 mM UDCA for an additional 1, 6, or 24 h. The macrophages of LPS alone group were treated with 1 μg/mL LPS for 1, 6, or 24 h. Cell pellets were subjected to a qRT-PCR analysis to detect the expression levels of (A) TNF-α, (B) IL-1α, (C) IL-1β (D) IL-6, and (E) IL-10 mRNA. The levels of each mRNA expression were normalized to the expression of GAPDH mRNA. The fold ratio of the 1 h control group was set at 1-fold and fold change was relatively calculated. Results are mean ± SD of triplicate experiments: *p < 0.05 and **p < 0.01, significant difference as compared to the control and to each other at the time point of 1, 6, or 24 h.

Fig 3 indicates that the 1 mM UDCA sample decreased significantly the number of inflammatory cytokines in LPS-stimulated RAW 264.7 macrophages in mRNA levels. Specifically, the expressions of IL-1α, IL-1β, and IL-6 were dramatically reduced by the 1 mM UDCA treatment at 6 h (Fig 3B–3D). In addition, unlike UDCA group, UDCA containing LPS group significantly increased the levels of anti-inflammatory cytokine IL-10 for 24 h (Fig 3E).

### Effect of UDCA on inflammatory and anti-inflammatory cytokines in protein levels in LPS-stimulated RAW 264.7 macrophages

**Fig 4:**
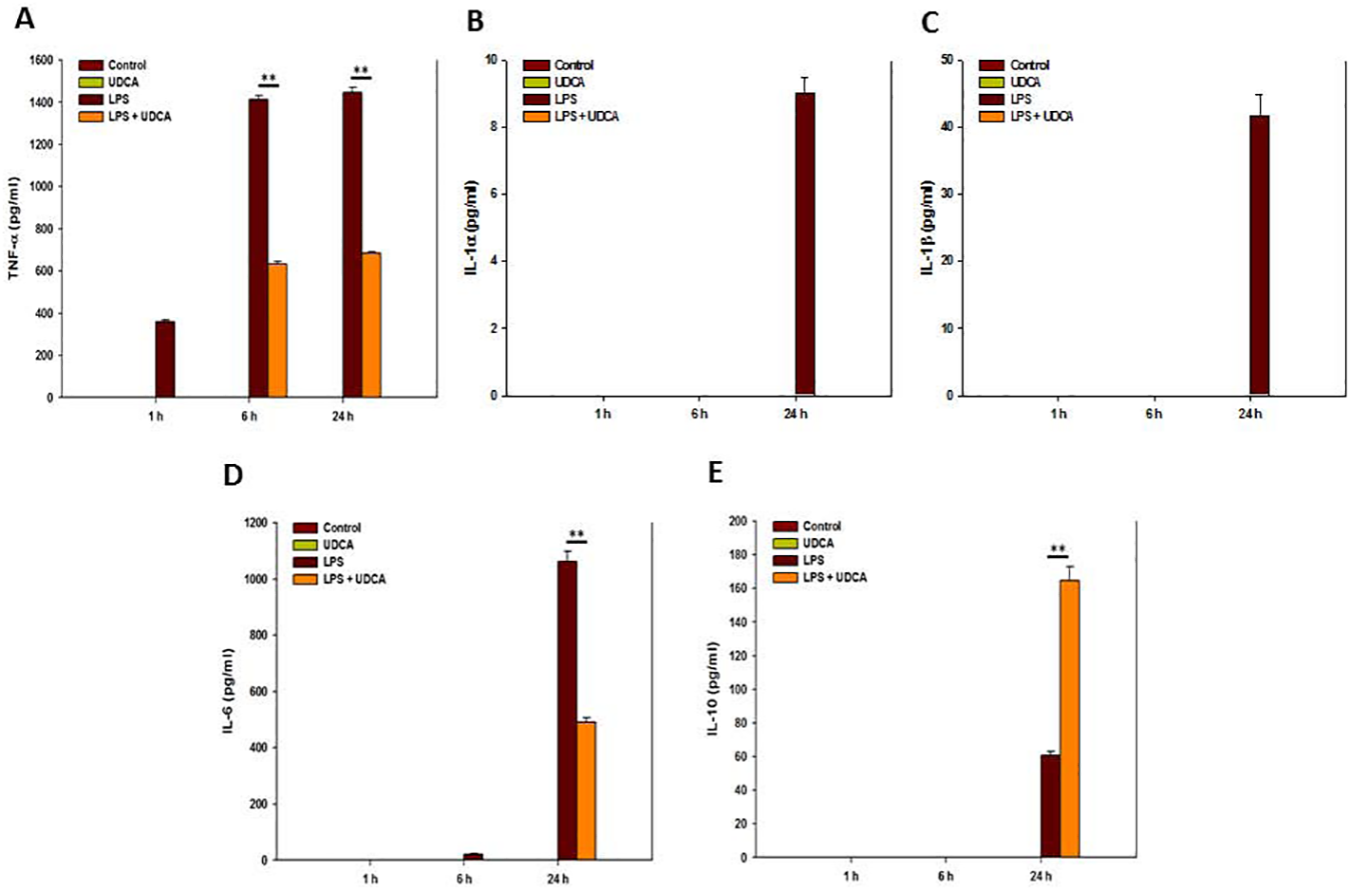
The protein expression in RAW 264.7 macrophages treated with UDCA (1 mM), LPS (1 μg/mL), or LPS (1 μg/mL) containing 1 mM UDCA for 1, 6, or 24 h. The macrophages in the UDCA with or without LPS group were pretreated with 1 mM UDCA for 1 h before the zero time point. Then, the UDCA alone group treated with 1 mM UDCA for an additional 1, 6, or 24 h and UDCA containing LPS group treated with LPS (1 μg/mL) containing 1 mM UDCA for an additional 1, 6, or 24 h. The macrophages of LPS alone group were treated with 1 μg/mL LPS for 1, 6, or 24 h. The collected supernatants were subjected to the plate of ELISA kit to detect the protein of (A) TNF-α, (B) IL-1α, (C) IL-1β (D) IL-6, and (E) IL-10. Results are mean ± SD of triplicate experiments: *p < 0.05 and **p < 0.01, significant difference as compared to the control and to each other at the time point of 1, 6, or 24 h.

The 1 mM UDCA sample decreased significantly the number of inflammatory cytokines in LPS-stimulated RAW 264.7 macrophages in protein levels (Fig 4A–4D). TNF-α was expressed at 1 h by LPS (Fig 4A) and the other cytokines were mostly expressed at 24 h (Fig 4B–4E). Even though inflammatory cytokines such as IL-1α, IL-β, and IL-6 recorded the maximum index at 24 h by LPS, UDCA significantly decreased the expression of the inflammatory cytokines at 24 h. On the other hand, UDCA containing LPS increased the anti-inflammatory cytokine IL-10 at 24 h (Fig 4E).

### Effect of UDCA on ERK, JNK, and p38 in mitogen-activated protein kinases (MAPKs) and IκBα phosphorylation in LPS-stimulated RAW 264.7 macrophages

**Fig 5:**
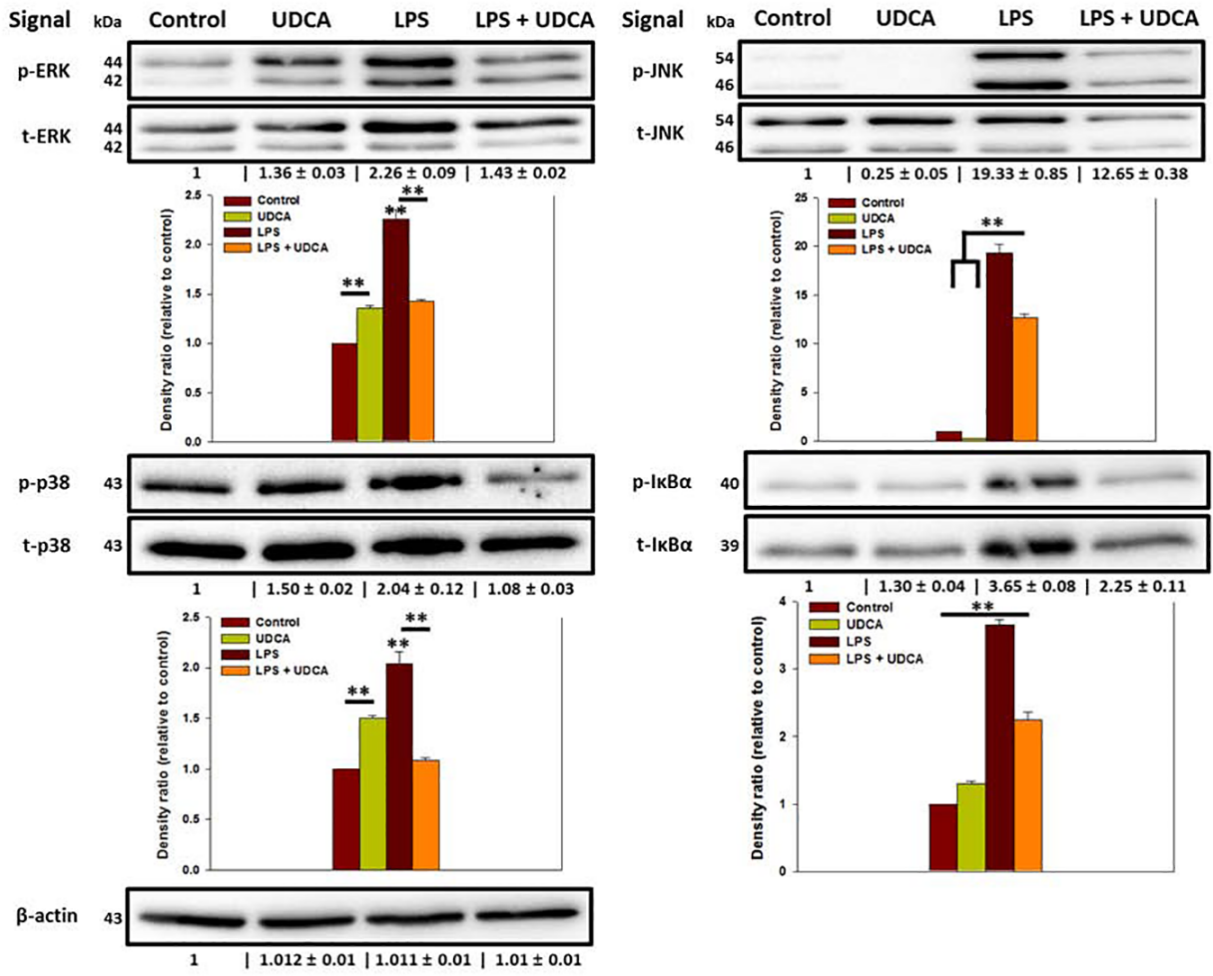
Effect of UDCA on the phosphorylation of ERK, JNK, p38 and IκBα in LPS-stimulated RAW 264.7 macrophages. The macrophages in the UDCA with or without LPS group were pretreated with 1 mM UDCA for 1 h before the zero time point. Then, the UDCA alone group treated with 1 mM UDCA for an additional 24 h and UDCA containing LPS group treated with LPS (1 μg/mL) containing 1 mM UDCA for an additional 24 h. The macrophages of LPS alone group were treated with 1 μg/mL LPS for 24 h. Immunoblotting was used to detect the phosphorylation form or the total form of ERK, JNK, p38, and IκBα in lysates prepared from the macrophages. β-actin was used as an internal control. The phosphorylation/total form (p/t form) volumes were calculated. The p/t form volume at control group was set at 1-fold and the ratio of the normalized fold change was relatively calculated and quantified. Results are mean ± SD of triplicate experiments: *p < 0.05 and **p < 0.01, significant difference as compared to the control and to each other for 24 h.

The LPS increased the p/t volume of ERK up to 2.26 ± 0.091. 1 mM UDCA significantly decreased the index to 1.43 ± 0.02 at 24 h (Fig 5). The p/t volumes of JNK and p38 were significantly decreased from 19.33 ± 0.85 to 12.646 ± 0.38 and from 2.04 ± 0.12 to 1.08 ± 0.03 at 24 h, respectively (Fig 5). The p/t volume index of IκBα was also significantly decreased from 3.65 ± 0.08 to 2.25 ± 0.11 at 24 h (Fig 5).

The mean values of the internal control, β-actin, were 1.012 ± 0.01 (UDCA group) 1.011 ± 0.01 (LPS group) and 1.01 ± 0.01 (LPS containing UDCA group).

## Discussion

In this study, the NO, qRT-PCR, and ELISA results constantly demonstrated that the 1 mM UDCA group inhibited the inflammatory response in LPS-stimulated RAW 264.7 macrophages without cytotoxicity. Anti-inflammatory effects can arise due to the suppression of the phosphorylation of the ERK, JNK, p38 and nuclear factor kappa B (NF-κB) signal pathways.

### Cytotoxicity of UDCA in RAW 264.7 macrophages

To determine the cytotoxicity concentration of UDCA, RAW 264.7 macrophages were treated with UDCA at various concentrations ranging from 0 mM to 5 mM for 24 h and CCK-8 assays were conducted for quantitative measurements (Fig 1B). Cell viability, above 80%, was regarded as evidence of a non-toxic density [22], and we found that UDCA did not demonstrate cytotoxicity up to 1 mM UDCA (Fig 1B).

### Effect of UDCA on LPS-stimulated NO secretion in RAW264.7 macrophages

The principal pro-inflammatory mediator, NO, is secreted in LPS-stimulated RAW 264.7 macrophage cells. Despite the fact that 1 mM UDCA increased NO secretion somewhat for 24 h (Fig 2A), the concentration significantly decreased NO secretion at 24 h in LPS-stimulated RAW 264.7 macrophages (Fig 2B). In particular, NO secretion was sharply increased at 24 h in the 2 mM UDCA group (Fig 2A). Joo et al. showed that UDCA effectively inhibited NO secretion at 100 μg/mL in LPS-stimulated cells and the density was recorded low index than that of control group [18]. The differences of UDCA densities and treated cell species (primary cultured rat microglial cells) may affect the results. In our study, considering cell viability results and the NO effect of UDCA alone and UDCA containing LPS groups, 1 mM UDCA was the most effective dose.

### Effect of UDCA on the production of inflammatory and anti-inflammatory cytokines in LPS-stimulated RAW 264.7 macrophages

For the evaluation of the anti-inflammatory effect of 1 mM UDCA in mRNA and protein levels on LPS stimulated RAW 264.7 macrophage cells, qRT-PCR and ELISA were conducted. For 24 h, UDCA constantly suppressed the expression of the LPS-stimulated pro-inflammatory cytokines such as TNF-α IL-1α, β, and IL-6 [23–25] (Fig 3A–3D). In addition, UDCA increased anti-inflammatory cytokine, IL-10, expression levels for 24 h (Fig 3E) [26]. Joo et al. also demonstrated that 100 μg/mL UDCA suppressed the IL-1β mRNA expression of Aβ-stimulated the microglia cells [18].

We found that 1 mM UDCA significantly inhibited inflammatory cytokines in protein levels using ELISA. While all the cytokines were recorded the maximum levels at 6 h in mRNA (Fig 3), IL-1α and β were not detected at 6 h in protein levels (Fig 4B and 4C). However, all the inflammatory cytokines showed the maximum levels at 24 h and UDCA significantly inhibited the inflammatory cytokines at 24 h. These results mean that it takes times from mRNA levels to protein levels for detection. In the Joo et al. study, IL-1β proteins were detected at 6 h. The differences of UDCA density and cell origin may affect the results [18].

In this study, protein levels were detected later than mRNA levels (Figs 3 and 4). There can be several reasons to explain this time difference between mRNA and protein expression. First, sensitive and reliable quantification of specific proteins demand specific antibodies of high affinity, whereas mRNA may be quantified very sensitively by qRT-PCR using gene-specific oligonucleotide primers [27]. Second, mRNA is relatively short-lived and is reached at instant steady-state concentration, whereas the concentration of protein in the medium is a result of the accumulation of protein [27]. Further studies are warranted to elucidate the exact reasons of time difference.

### Effect of UDCA on ERK, JNK, and p38 in MAPKs and IκBα phosphorylation in LPS-stimulated RAW 264.7 macrophages

LPS is a well-known for the stimulator of MAPKs. ERK, JNK, and p38 signal pathways in the MAPKs regulate the inflammatory responses [28–30]. These signals are phosphorylated by LPS [30]. NF-κB is also a major regulator in pro-inflammatory signal pathways [9]. NF-κB exists in the cytoplasm as inactivated form with the inhibitory protein IκBα. However, when IκBα is phosphorylated by the inflammatory stimuli, NF-κB is translocated to nucleus for inflammatory downstream [31]. In this study, 1 µg/ml LPS significantly stimulated the phosphorylation of pro-inflammatory signals, then 1 mM UDCA significantly inhibited the stimulated phosphorylation. These results demonstrate that UDCA affects the ERK, JNK, p38 and NF-κB pathways.

The evaluation of UDCA as anti-inflammatory drug in *in vivo* levels was studied by Kullmann et al. [32]. The authors showed that 10 mg/kg of UDCA can attenuate the acute intestinal inflammation of Sprague Dawley rat models.

Although we did not conduct an *in vivo* study using LPS model, to the best of our knowledge, this study is the first to show that UDCA inhibits the pro-inflammatory response and increases the anti-inflammatory effect in LPS-stimulated RAW 264.7 macrophages through qRT-PCR and western blot assay.

## Conclusion

UDCA inhibits LPS-stimulated pro-inflammatory response in RAW 264.7 macrophages. UDCA suppresses phosphorylation of ERK, JNK, and p38 in MAPKs. UDCA also inhibits phosphorylation of IκBα. These results suggest that UDCA can serve as a useful anti-inflammatory drug.
